# Photobiomodulation laser therapy in a Lenvatinib-related osteonecrosis of the jaw: A case report

**DOI:** 10.4317/jced.58323

**Published:** 2021-06-01

**Authors:** Luis Monteiro, Catarina Vasconcelos, José-Júlio Pacheco, Filomena Salazar

**Affiliations:** 1 Oral Medicine and Oral Surgery Department, University Institute of Health Sciences (IUCS), Gandra 4585-116, Portugal; 2 Cancer Research Group, CESPU, Instituto de Investigação e Formação Avançada em Ciências e Tecnologias da Saúde (IINFACTS), University Institute of Health Sciencies (IUCS), Gandra 4585-116, Portugal; 3 Oral Diseases Research Group, CESPU, Instituto de Investigação e Formação Avançada em Ciências e Tecnologias da Saúde (IINFACTS), University Institute of Health Sciencies (IUCS), Gandra 4585-116, Portugal

## Abstract

**Background:**

Medication-related osteonecrosis of the jaw (MRONJ) is a serious adverse drug reaction often presenting as a post-surgery complication that may interfere in the quality of the patient’s life. In the last decade, additionally to bisphosphonates, other drugs have been associated with MRONJ including other antiresorptive, antiangiogenic or multitarget drugs such as levantinib. The data on MRONJ associated to lenvatinib is scarce with no guidelines for best management option. Our aim is to report a case of MRONJ associated with lenvatinib and the useful of a non-invasive management using local photobiomodulation (PBM) therapy with a 635nm diode laser.

**Material and Methods:**

A 61-year-old female patient with a follicular thyroid carcinoma (stage IV) and taking lenvatinib presented to our Oral Medicine Unit with a painful non-healing ulcer with bone exposure, in the posterior right maxilla, after an extraction of a molar tooth which occurred 4 months previously. Bone rarefaction was detected in CT scan in the same affected area. We diagnosis a lenvatinib-related osteonecrosis of the Jaw (LRONJ). We performed 5 sessions of PBM treatment using a 635 nm diode laser, delivering 10J/ cm2 in affected area.

**Results:**

At the end of the first session, a relief in the pain was already refereed by the patient. One month after, the oral mucosa was completely healed and tissue integrity was confirmed clinically and on panoramic radiograph and the patient referred an increase in her quality of life. On the last follow up after 6 months the patient was without any recurrence.

**Conclusions:**

A lenvatinib-related osteonecrosis of the maxilla in a female patient is reported here for the first time. Moreover, a non-invasive management using PBM laser therapy has shown a successful healing of involved tissues and immediate symptoms relief improving the quality of life of the patient.

** Key words:**Lenvatinib, MRONJ, osteonecrosis of the jaw, tyrosine kinase inhibitors, photobiomodulation.

## Introduction

Medication-related osteonecrosis of the jaw (MRONJ) is now a well-known adverse drug reaction that affect the maxillary bones and can infer seriously in the patient’s quality of life (1,2). MRONJ has been defined by the American Association of Oral and Maxillofacial Surgery (AAOMS) as exposed bone or bone that can be detected through a extraoral or intraoral fistula, in the maxillofacial area, can persist for 8 or more weeks, occurring in patients which are or were in treatment with anti-angiogenic or antiresorptive drugs and in the absence of a previous radiation treatment or metastatic disease to the jaws (1). Osteonecrosis of the jaws (ONJ) can affect both jaws but typically affects the mandible. Clinically the lesion may cause swelling, discomfort, pain, infection, or even pathologic fracture (1,3-5).

The biopathology of MRONJ is not fully understood, however often reported as related with a knockdown of bone remodelling and impaired angiogenesis (1,6). The existence of local risk factors that contribute or triggers MRONJ are reported with invasive dental procedures as the main cause (including tooth extraction as the precipitating event in more than 50% of the cases) (1).

Since the primary association with bisphosphonates on the first cases other drugs have been related with an increased risk of inducing osteonecrosis of the jaw´s including other antiresorptive drugs such as denosumab, tumour necrosis factor-alpha (TNF-) inhibitors (e.g. etanercept, adalimumab, infliximab), mammalian target of rapamycin (mTOR) inhibitors (e.g., everolimus), antiangiogenic drugs (e.g., bevacizumab) or other receptor tyrosine kinase inhibitors (RTKI) (e.g. sunitib, imatinib) sometimes directed to multiple targets such as lenvatinib (3,7-9). The association of lenvatinib with MRONJ is scarce on literature, providing no information on prevalence, clinical presentation or prognosis of potential MRONJ (9). Moreover, there is no data on management of lenvatinib–related osteonecrosis of the jaw´s (LRONJ), as for example using conservative approach (e.g. laser photobiomodulation) or even invasive treatments. Using the CARE list guidelines for reporting case reports, the aim of this work is to report a case of a maxillary osteonecrosis associated with lenvatinib and the useful of a non-invasive management with photobiomodulation (PBM) therapy with a 635nm diode laser.

## Case Report

**Figure 1:**
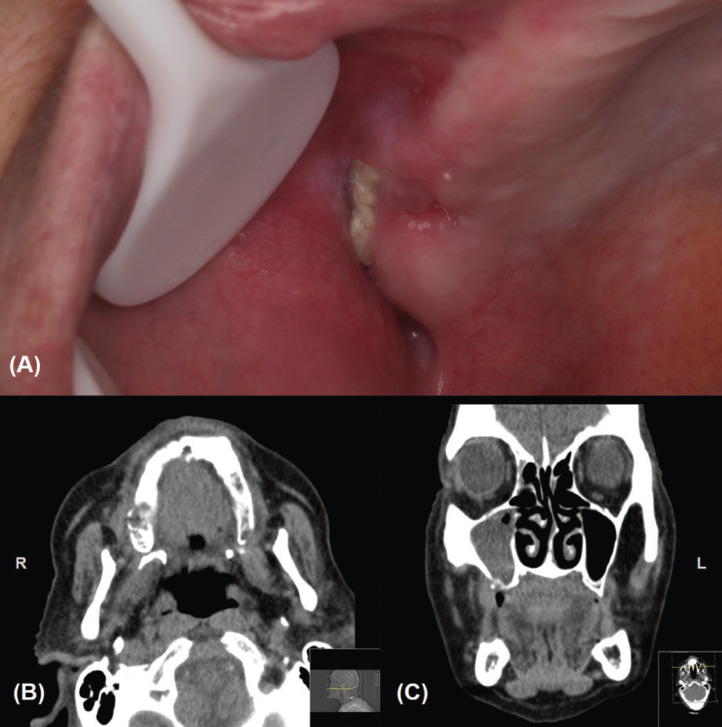
Clinical presentation of a non-healing ulcer with bone exposure compatible with a lenvatinib-related osteonecrosis of the maxilla (A); CT scan showing bone rarefaction of maxillary posterior and right alveolar process (B and C).

A 61-year-old female patient was referred to our Oral Medicine Unit at the CESPU Parcerias Clinic, send by the GDP colleagues because of a painful non-healing socket in the maxilla after an extraction of a carious tooth. During the anamnesis, the patient referred that she felt an increasing amount of oral discomfort, drainage of fluids, and facial right side pain after the dental extraction of the posterior molar of right maxilla four months before. An intense pain on right and upper side of the mouth was described by the patient in the last weeks (VAS scale – 8). Her medical antecedents included a follicular thyroid carcinoma in stage IV stage with bony metastases (not in the craniofacial area) and submitted to total thyroidectomy, two years before. Antineoplastic therapy included lenvatinib 24mg orally once a month. She had performed palliative radiotherapy (not including jaws) for bone metastases. She did not take bisphosphonates or denusomab and reported no other important medications or allergies to any medication. On the contact with the GDP, a conservative approach was followed using local antiseptic agents (chlorhexidine rinses 0.2%, three times daily) and systemic antibiotic therapy (amoxicillin plus clavulanic acid, 875+125mg, two times daily) since the non-healing and bone exposure detection, although without any improvement in pain and healing. During the clinical examination, we observed a bone exposure in the posterior right maxilla with a 1.5cm of biggest size (Fig. 1A), with intense pain to palpation of adjacent tissues. We performed computed tomography (CT) scan that showed a bone density loss in the right and posterior area of the maxilla compatible with an area of osteonecrosis (Fig. 1B,C). Additionally, we observed also an almost full-filling of the right maxillary sinus. No associated neck lymphadenopathy was detected. Neoplastic disease was appropriately ruled out at this location. In the view of these observations we performed a diagnosis of a medication-related osteonecrosis of the maxilla associated with lenvatinib, grade III according to the AAOMS, 2014 (1), with a questionable prognosis.

**Figure 2:**
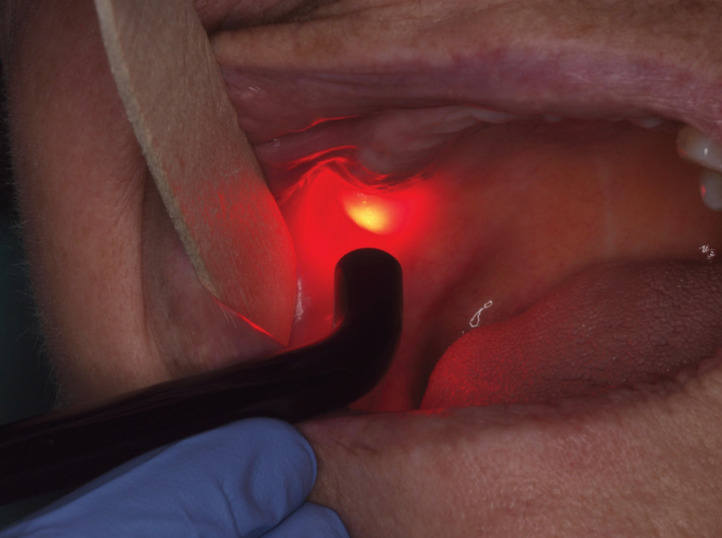
Application of handpiece for photobiomodulation at the affected area of LRONJ.

**Figure 3:**
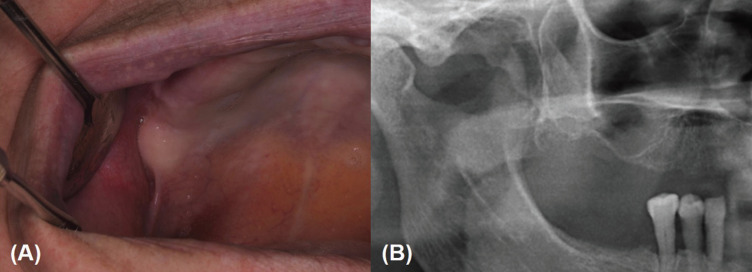
Complete healing of mucosal alveolar mucosa (A) and bone integrity on panoramic radiograph (B) in a follow-up of 6 months after the end of the treatment sessions.

After informed consent we performed a PBM treatment of the affected area using a 635 nm wavelength diode laser (Lasotronix®, Diode Laser DiodeLX model SMART M, Żytnia, Piaseczno, Poland). We performed 5 sessions during 5 consecutive weeks (one session per week) using an 8-mm cylindrical applicator handpiece, in a continuous mode for 25 seconds, delivering 10 J/cm2 in affected area (Fig. 2). All safety measures for protecting the patient, operator, and assistant were followed. After the first session the patient reported an immediate relief in the treated area (VAS scale – 1). At the end of last session (~1 month after the beginning of treatment) the oral mucosa was completely healed and bone integrity was confirmed on panoramic radiograph. The patient refereed any symptom since performing the laser sessions (including pain that corresponded to 0 in VAS scale since the 2nd week of treatment) referring also an increase in her quality of life. On the last follow up the patient was without recurrence after 6 months of follow-up (Fig. 3).

## Discussion

In the last decade, a wide spectrum of drugs has been described as related with osteonecrosis of the jaws which has led to the actual designation of MRONJ. However, few cases of osteonecrosis related with the family of RTKIs are reported and there are exceedingly rare cases of osteonecrosis associated with lenvatinib (3,9). We report here a conservative management of a LRONJ using PBM laser therapy with successful healing of involved tissues and immediate symptoms relief in a female patient with an advanced and metastatic follicular thyroid carcinoma.

Lenvatinib is a novel multitarget drug from the family of RTKI, directed mainly to vascular endothelial growth factor receptors, but also fibroblast growth factor receptors (FGF), platelet-derived growth factor receptor-alpha (PDGF), RET and KIT proto-oncogenes, showing antiangiogenic properties and potent anti-tumour activity. It is often used in treatment of advanced thyroid cancer, renal cell carcinoma and hepatocellular carcinoma (10,11). Possible side effects of this drug includes hepatotoxicity, cardiac dysfunction, arterial thromboembolic events, renal failure and amongst others wound healing complications (10,11). In our case, we confirm a mucosal non-healing and exposure of bone with more than 3 months, with a bone rarefaction observed in the CT scan, and without reports of other medication that could be related with osteonecrosis (except for lenvatinib) or radiotherapy including maxillary area. This let us to diagnose an LRONJ. Mauceri *et al*. (9) reported a LRONJ in an Italian man with an Hurthle cell thyroid cancer also located in a maxilla bone, with a fistula, purulent exudation and chronic pain.

PBM has been suggested to be a promising modality of treatment for patients with MRONJ in a single treatment or as an adjunct treatment with some studies reporting the beneficial effect of this method for these patients (4,12,13) including improve healing activity and reduce of pain as we observed in our patient. This has been reported in ONB patients also by the group of Romeo *et al*. (12). Moreover, during the initial period of four months only performing antibiotic therapy, any improvement of the lesion and symptomology was seen. Interestingly, additionally to the reduction of pain already refereed at the first sessions of PBM, at the end of the last session the mucosae were completed closed, which suggest the contribution of PBM for healing process, perhaps as a “gamechanger” in these lesions. No complications or side effects were observed. Also there was no need of invasive methods which is a real advantage for these patients many times with advanced disease status. PBM can influence many important biological processes including the activation of several cellular cascade events such as production of mitochondrial adenosine triphosphate and intracellular Ca++, with consequent improve of cell energy metabolism, an increase in blood supply with potential improve on healing of the tissues. PBM has shown also to have analgesic and anti-inflammatory properties, increasing the release of beta-endorphins and reducing the amount of inflammatory cytokines including bradykinin, and prostaglandins which leads to a decrease inflammation, oedema and pain as also decontamination properties (14). This case reinforces also the importance of knowledge and awareness of the risk factors for osteonecrosis of the patients that presents to our clinics for oral treatments including local factors such as surgical procedures as also systemic factors and drug related factors specially for less common drugs related to MRONJ such as lenvatinib. (15). We acknowledge some limitations on this report, as naturally the power limitation of a single case report. However, to our knowledge, this is the first report of a LRONJ in a female patient with a successful conservative management using PBM laser therapy and without any secondary effects. Although we recognise that we could not prove with a single case report that the observed healing was due only to PBM treatment, we could clearly observe an elimination of pain after the laser treatment and improve the quality of life of the patient in this LRONJ.

In conclusion, we report a case of an lenvatinib-related osteonecrosis in a maxilla of a female patient with a metastatic follicular thyroid carcinoma, contributing for the worldwide awareness and knowledge of the potential effects of multitarget drugs from the RTKI family, in special lenvatinib, as risk factors of a MRONJ. Moreover, we show the usefulness of a non-invasive management using PBM laser therapy with successful healing of involved tissues and immediate symptoms relief improving the quality of life of the patient.
